# Factors related with colorectal and stomach cancer screening practice among disease-free lung cancer survivors in Korea

**DOI:** 10.1186/s12885-017-3583-z

**Published:** 2017-08-30

**Authors:** Sang Min Park, Jongmog Lee, Young Ae Kim, Yoon Jung Chang, Moon Soo Kim, Young Mog Shim, Jae Ill Zo, Young Ho Yun

**Affiliations:** 1 Department of Biomedical Science, Seoul National University College of Medicine, 103 Daehak-ro, Jongno-gu, Seoul, 110-799 Republic of Korea; 2 Department of Family Medicine, Seoul National University College of Medicine, Seoul, Republic of Korea; 3 Center for Lung Cancer, National Cancer Center, Goyang, Republic of Korea; 4 National Cancer Control Institute, National Cancer Center, Goyang, Republic of Korea; 5 Lung and Esophageal Cancer Center, Samsung Comprehensive Cancer Center, Samsung Medical Center, Seoul, Republic of Korea; 6 Cancer Research Institute, Seoul National University College of Medicine, Seoul, Republic of Korea

**Keywords:** Colorectal cancer screening, Stomach cancer screening, Lung cancer survivor, Physician recommendation

## Abstract

**Background:**

Lung cancer survivors are more likely to develop colorectal and stomach cancer than the general population. However, little is known about the current status of gastrointestinal cancer screening practices and related factors among lung cancer survivors.

**Methods:**

We enrolled 829 disease-free lung cancer survivors ≥40 years of age, who had been treated at two hospitals from 2001 to 2006. The patients completed a questionnaire that included stomach and colorectal cancer screening after lung cancer treatment, as well as other sociodemographic variables.

**Results:**

Among lung cancer survivors, correlations with stomach and colorectal screening recommendations were 22.7 and 25.8%, respectively. Of these, 40.7% reported receiving physician advice to screen for second primary cancer (SPC). Those who were recommended for further screening for other cancers were more likely to receive stomach cancer screening [adjusted odds ratios (aOR) = 1.63, 95% confidence interval (CI), 1.16–2.30] and colorectal cancer screening [aOR = 1.37, 95% CI, 0.99–1.90]. Less-educated lung cancer survivors were less likely to have stomach and colorectal cancer screenings.

**Conclusions:**

Lack of a physician’s advice for SPC screening and lower educational status had negative impact on the gastrointestinal cancer screening rates of lung cancer survivors.

**Electronic supplementary material:**

The online version of this article (10.1186/s12885-017-3583-z) contains supplementary material, which is available to authorized users.

## Background

Although advanced stage lung cancer has a poor prognosis, [1] early stage lung cancer can be treated with surgical resection, resulting in an improved prognosis [2, 3]. Recently, the US Preventive Services Task Force (USPSTF) recommended annual screening for lung cancer, using low dose computed tomography (CT) for individuals at a high risk for this disorder [4]. Furthermore, the clinical practice of low dose CT scanning as an early detection tool, as well as advances in cancer treatment, could lead to an increased number of lung cancer survivors [3, 4].

Previous studies have reported that lung cancer patients were at an increased risk for second primary cancers (SPCs) [5, 6]. For second primary gastrointestinal cancers, a recent study reported that early stage lung cancer patients had approximately a 40% increased risk of colorectal and stomach cancer than the general population [6]. The Global Burden of Disease Study in 2017 has demonstrated that colorectal cancer and stomach cancer are ranked within global top 5 cancers, [7] colorectal cancer screening and stomach cancer screening are introduced in several countries [8–10]. Cancer survivors were recommended to adhere routine age- and sex-appropriate cancer screening guideline in general population [11–13]. Especially, as colorectal cancer is the most common cancer, and stomach cancer remains the second common cancer in Korea, [14] continued surveillance program regarding gastrointestinal cancer screening for Korean lung cancer survivors will be needed. However, little is known about the gastrointestinal cancer screening practices in lung cancer survivors. The aim of our survey was to determine the patterns of screening for colorectal and stomach cancer screening and related factors in lung cancer survivors who were disease free in Korea. We hypothesized that not only low social-demographic status but also lack of physicians’ advice for SPC screening or patients’ misperception about their risk of SPC would have negative impacts on the gastrointestinal cancer screening behaviors in lung cancer survivors.

## Methods

### Study participants

We identified 2049 patients who had been treated for lung cancer in two hospitals in the Republic of Korea, between 2001 and 2006. We performed a cross-sectional survey of lung cancer survivors in 2007. Eligible subjects were contacted by telephone, and those who agreed to participate were surveyed with questionnaires at home or at the clinic. Lung cancer survivors who were treated with curative surgery and had no other history of cancer were eligible to participate. The institutional review board of the National Cancer Center, Korea reviewed and approved the protocol of our study. Details of the study design have been previously described [15].

### Definition of appropriate uptake of gastrointestinal cancer screening

For stomach cancer screening, Korean National Cancer Screening Program (KNCSP) [8] recommended gastroscopy or double-contrast upper gastrointestinal series every 2 years for general population ≥ 40 years of age, and the Japanese government introduced gastroscopy as a national screening program [9, 16]. For early detection of colorectal cancer, annual FOBT was recommended for those ≥50 years of age by the KNCSP [8] and by the USPSTF. The American Cancer Society (ACS) has recommended sigmoidoscopy every 5 years, a double-contrast barium enema every 5 years, or a colonoscopy every 10 years [10, 17, 18]. However, colorectal screening guidelines for the general population could underestimate the actual needs of cancer survivors. One previous study reported that for cancer survivors aged 40-years-old, colonoscopy every 5 years might be an economically feasible strategy [19]. As a baseline analysis of colorectal cancer screening, we considered all the above mentioned recommendations to be compliant with colorectal screening among lung cancer survivors ≥40 years of age. We also performed sensitivity analysis with subject ≥50 years of age, using the above cancer screening recommendations.

To assess the practices of stomach and colorectal cancer screening after cancer treatment, lung cancer survivors were asked the following questions (Additional file 1): 1) “When did you receive a gastroscopy or double-contrast upper gastrointestinal series recently?” with responses of “no,” “≤ 2 years ago,” “2–5 years ago,” and “>5 years ago”; 2) “What kind of colorectal cancer screening test did you receive?” with responses of “no,” “fecal occult blood test (FOBT),” “double-contrast barium enema,” “sigmoidoscopy,” and “colonoscopy”; and 3) “If you receive a colorectal cancer screening, when did you receive the last colorectal cancer screening test?” with responses of “<1 year ago,” “1–5 years ago,” “5–10 years ago,” and “>10 years ago.”

### Independent variables

Lung cancer survivors were asked to approximate their risk of SPC compared with cancer risk in general population, with the responses being lower, similar, or higher. The survey also included question about receiving a physicians’ recommendation to screen for SPC after lung cancer treatment. In addition, participants were asked to answer questions about age, highest educational attainment, ethnicity, income, health behavior (physical activity, smoking, alcohol consumption, height and weight), and health-related quality of life (the European Organization for Research and Treatment of Cancer Quality of Life Questionnaire Core-30 item and lung cancer module, Hospital Anxiety and Depression Scale and Posttraumatic Growth Inventory) through our systematically organized questionnaire. From the hospital cancer registries, we gathered information about clinical characteristics such as ages at cancer diagnosis, tumor stage, type of surgery, history of chemotherapy or radiotherapy, and recurrence.

### Statistical analysis

Descriptive statistics were reported for each response. Among subjects, those who received gastroscopy or double-contrast upper gastrointestinal series within 2 years were defined as lung cancer survivors with appropriate stomach cancer screening [8]. Lung cancer survivors who received FOBT within 1 year, a double-contrast barium enema within 5 years, sigmoidoscopy within 5 years, or colonoscopy within 10 years were defined as receiving appropriate colorectal cancer screening [10, 17, 18]. We then calculated the occurrences of lung cancer survivors who had second gastrointestinal cancer screening according to these guidelines.

Adjusted odds ratios were determined by logistic regression analysis, main independent variable being physicians’ advice for SPC screening, perception of second cancer risk, highest educational attainment, and family income adjusted for age, stage, marital status, smoking status, and alcohol consumption. We also performed sensitivity analysis with lung cancer survivors ≥50 years of age. All statistical analyses were two-sided and performed using STATA 10.0 software (Stata Corp., College Station, TX, USA). The significance level was set at *P* < 0.05.

## Results

Among the potentially eligible population, 126 (6.1%) had died, 290 (14.2%) could not be contacted in spite of multiple attempts. Excluded from this study were patients whose cancer had recurred at the time of the survey. All participants provided written informed consent. Of the 1633 contacted patients, 727 (35.5%) refused to participate, and 906 (44.2%) consented to participate. Among the respondents, 76 patients had cancer which had recurred, or were receiving cancer therapy at the time. One subject <40 years of age was excluded. The analysis included 829 lung cancer survivors ≥40 years of age.

The mean age of 829 lung cancer survivors was 62.9 years (40–78 years). Of these, 44.2% had no more than a 6th grade education, and 63.1% was diagnosed as stage I lung cancer. Among disease-free lung cancer survivors, 40.7% reported receiving physician advice to screen for other cancers. About one out of ten reported a perception that they had a lower risk of other cancers than the general population, and 60.1% believed that they had a higher risk of other cancers than general population (Table 1). When we compared the characteristics of the participants and non-participants among1633 contacted patients, responders were more likely to be men and to live in metropolitan areas than non-participants (Additional file 2: Table S1).

**Table 1:** Demographic and clinical characteristics of disease-free lung cancer survivors

Characteristics	N	%
Age, years (mean)	(62.9)	
40–49	58	7.0
50–64	379	45.7
≥ 65	393	47.3
Gender		
Male	637	76.8
Female	193	23.2
Marital Status		
Married	764	90.7
Unmarried, divorced, or bereaved	66	9.3
Level of Education		
≤ 6 years	229	44.2
7–11 years	389	28.6
≥ 12 years	210	27.2
Monthly household income, $(US)		
≥ 3000	225	27.2
1000–2999	363	43.8
< 1000	240	29.0
Stage		
I	519	63.1
II	173	21.0
III	131	15.0
Receiving physician advice to screen for SPC		
Yes	337	40.7
No	491	59.3
Self-perception of the SPC risk		
Lower than the general population	77	9.3
Same as the general population	253	30.6
Higher than the general population	496	60.1

*SPC* second primary cancer

**Fig. 1:**
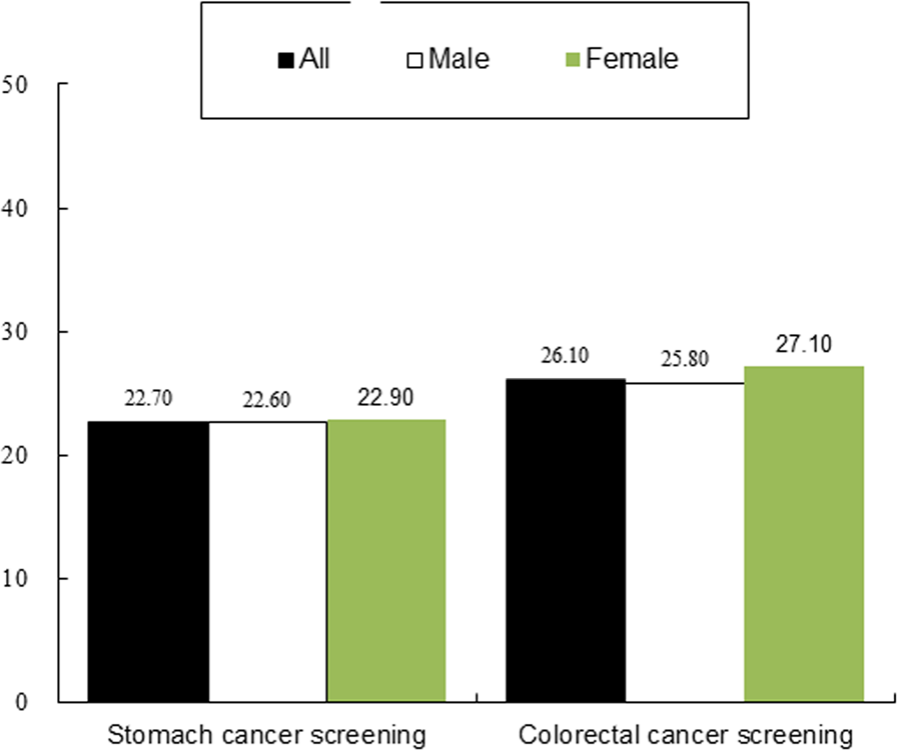
Percentage of lung cancer survivors who received stomach^a^ and colorectal^b^ cancer screening. ^a^Among lung cancer survivors, those who received gastroscopy or double-contrast upper gastrointestinal series within 2 years were defined as lung cancer survivors with appropriate stomach cancer screening. ^b^Lung cancer survivors who received FOBT within 1 year, a double-contrast barium enema within 5 years, sigmoidoscopy within 5 years, or colonoscopy within 10 years were defined as receiving appropriate colorectal cancer screening

The proportions for receiving appropriate stomach cancer screening and colorectal cancer screening were 22.7 and 26.1%, respectively (Fig. 1). Both male and female lung cancer survivors showed similar trends of SPC cancer screening.

### Factors related to the uptake of stomach cancer screening for lung cancer survivors

Lung cancer survivors who recalled being informed about the need for SPC screening were more likely to have stomach cancer screening in multivariate-adjusted analysis [adjusted OR (aOR) = 1.63, 95% CI, 1.16–2.30], and these associations were greater among male patients (Table 2). When we assessed compliance of stomach cancer screening practices by monthly household income and perception of SPC risk, there were no significant differences among the groups. Lung cancer survivors with the most education (≥ 12 years) were more likely to have stomach cancer screening (aOR = 1.72, 95% CI, 1.00–2.96), especially for male patients (aOR 1.87, 95% CI, 1.00–3.51). Multivariate analysis of patients ≥50 years of age showed associations between the above factors with uptake of stomach cancer screening that were similar to those of patients ≥40 years of age.

**Table 2:** Factors related to the uptake of stomach cancer screening^a^ for lung cancer survivors

Variables	All patients (*N* = 829)			Male patients (*N* = 641)			Female patients (*N* = 188)		
	%	Age-adjusted OR (95% CI)	Multivariate OR^b^ (95% CI)	%	Age-adjusted OR (95% CI)	Multivariate OR^b^ (95% CI)	%	Age-adjusted OR (95% CI)	Multivariate OR^b^ (95% CI)
Receiving physician advice to screen for SPC									
No	19.8	1.0	1.0	19.5	1.0	1.0	20.8	1.0	1.0
Yes	26.7	1.52 (1.09–2.11)	1.61 (1.14–2.26)	27.4	1.59 (1.09–2.32)	1.73 (1.17–2.56)	24.7	1.32 (0.66–2.63)	1.30 (0.61–2.80)
Perceived risk of SPC in lung cancer survivors									
Lower than general population (GP)	20.8	1.0	1.0	22.9	1.0	1.0	12.5	1.0	1.0
Same or higher than GP	22.6	1.07 (0.60–1.91)	1.16 (0.63–2.13)	22.4	0.95 (0.51–1.79)	0.97 (0.50–1.88)	23.0	1.89 (0.40–8.92)	2.34 (0.44–12.43)
Monthly household income, ($US)									
< 1000	19.6	1.0	1.0	18.9	1.0	1.0	22.0	1.0	1.0
1000–2999	21.8	1.11 (0.73–1.67)	1.03 (0.66–1.59)	22.5	1.20 (0.75–1.92)	1.06 (0.64–1.73)	19.5	0.79 (0.31–1.97)	1.10 (0.33–3.05)
≥ 3000	27.4	1.44 (0.91–2.23)	1.48 (0.94–2.32)	27.2	1.50 (0.88–2.56)	1.20 (0.66–1.71)	28.1	1.24 (0.48–3.21)	1.34 (0.37–4.95)
Education									
≤ 6 years	17.5	1.0	1.0	14.6	1.0	1.0	24.6	1.0	1.0
7–11 years	22.7	1.33 (0.86–2.06)	1.40 (0.88–2.22)	24.7	1.88 (1.11–3.19)	1.93 (1.11–3.35)	15.9	0.51 (0.22–1.20)	0.46 (0.17–1.24)
≥ 12 years	28.6	1.78 (1.10–2.87)	1.72 (1.00–2.96)	26.9	2.09 (1.18–3.72)	1.87 (1.00–3.51)	35.9	1.43 (0.56–3.64)	1.34 (0.40–4.48)

*OR* odds ratio, *CI* confidence interval, *SPC* second primary cancer

^a^Subjects who received gastroscopy or double-contrast upper gastrointestinal series within 2 years were defined as lung cancer survivors with appropriate stomach cancer screening

^b^Adjusted for age, stage, marital status, education, family income status, smoking status, alcohol consumption, receiving recommendation for other cancer screening, and the perception of secondary cancer risks

### Factors related to the uptake of colorectal cancer screening for lung cancer survivors

Participant’s reporting to receive a physicians’ advice to screen for other cancers was positively associated with receiving colorectal cancer screening in both age-adjusted analysis (aOR = 1.52; 95% CI, 1.09–2.12) and multivariate analysis (aOR = 1.37, 95% CI, 0.99–1.91; Table 3). Self-perception of SPC risk was not significantly associated with colorectal screening practices. Less-educated patients were less likely to have colorectal cancer screening (aOR = 1.76, 95% CI, 1.05–2.96). Family income was also significantly associated with colorectal cancer screening compliance among female lung cancer survivors. Compared with family income less than $1000/month, female lung cancer survivors with a higher income (≥ $3000/month) were more likely to undergo a colorectal cancer screening (aOR = 5.09, 95% CI, 1.28–20.14).

**Table 3:** Factors related to the uptake of colorectal cancer screening^a^ for lung cancer survivors

Variables	All patients (*N* = 829)			Male patients (*N* = 641)			Female patients (*N* = 188)		
	%	Age-adjusted OR (95% CI)	Multivariate OR^b^ (95% CI)	%	Age-adjusted OR (95% CI)	Multivariate OR^b^ (95% CI)	%	Age-adjusted OR (95% CI)	Multivariate OR^b^ (95% CI)
Receiving physician advice to screen for SPC									
No	23.3	1.0	1.0	22.9	1.0	1.0	25.0	1.0	1.0
Yes	29.0	1.52 (1.09–2.12)	1.37 (0.99–1.91)	29.2	1.38 (0.96–1.99)	1.46 (1.00–2.12)	28.2	1.30 (0.68–2.48)	1.14 (0.53–2.48)
Perceived risk of SPC in lung cancer survivors									
Lower than general population (GP)	21.6	1.0	1.0	22.0	1.0	1.0	20.0	1.0	1.0
Same or higher than GP	25.9	1.07 (0.60–1.91)	1.14 (0.65–2.03)	25.7	1.16 (0.62–2.16)	1.13 (0.59–2.15)	26.6	1.08 (0.32–3.62)	1.37 (0.33–5.63)
Monthly household income, $(US)									
< 1000	24.2	1.0	1.0	24.6	1.0	1.0	22.5	1.0	1.0
1000–2999	21.6	1.96 (0.73–1.67)	0.73 (0.48–1.12)	22.5	0.85 (0.55–1.32)	0.68 (0.43–1.10)	17.9	0.88 (0.44–4.48)	1.51 (0.47–4.88)
≥ 3000	34.5	1.45 (0.91–2.30)	1.22 (0.74–2.02)	32.2	1.47 (0.89–2.41)	1.02 (0.58–1.82)	40.7	2.98 (1.18–7.53)	5.09 (1.28–20.14)
Education									
≤ 6 years	20.8	1.0	1.0	17.8	1.0	1.0	28.6	1.0	1.0
7–11 years	25.3	1.33 (0.86–2.05)	1.34 (0.87–2.09)	27.4	1.73 (1.05–2.84)	1.91 (1.13–3.23)	17.7	0.56 (0.25–1.27)	0.34 (0.12–0.94)
≥ 12 years	33.2	1.78 (1.11–2.88)	1.76 (1.05–2.96)	30.3	2.14 (1.25–3.68)	1.87 (1.02–3.41)	48.3	2.75 (1.12–6.78)	1.35 (0.40–4.48)

*OR* odds ratio, *CI* confidence interval, *SPC* second primary cancer

^a^Subjects who received FOBT within 1 year, a double-contrast barium enema within 5 years, sigmoidoscopy within 5 years, or colonoscopy within 10 years were defined as receiving appropriate colorectal cancer screening

^b^Adjusted for age, stage, marital status, education, family income status, smoking status, alcohol consumption, receiving recommendation for other cancer screening, and perception of secondary cancer risks

When we performed a sensitivity analysis with subjects ≥50 years of age, male lung cancer survivors who received a physicians’ advice of screening for second cancers were more likely to have a colorectal cancer screening (aOR = 1.48, 95%CI, 1.00–2.18).

## Discussion

Our study showed that colorectal and gastric cancer screening practices among lung cancer survivors was less than optimal. In addition, half of these patients did not recall receiving advice from their physicians about SPC screening. Lack of a physicians’ advice for SPC screening and lower educational status might have negative impact on the gastrointestinal cancer screening rates of lung cancer survivors.

Because lung cancer survivors have an increased risk of colorectal and stomach cancer development, [5, 6] following the recommendations of gastrointestinal cancer screening for the average risk population should be needed at a minimum. However, our study showed that less than 30% of disease-free lung cancer survivors adhered to these colorectal and stomach screening recommendations. Several previous studies [20, 21] and one recent meta-analysis [22] reported that many cancer survivors did not receive screening tests recommended for the detection of SPCs, although cancer survivors received more frequent screening for cancers than non-cancer controls. These findings emphasized the need to identify effective methods to increase cancer screening practices for cancer survivors. Several interventions, such as reminders, small media, and face-to-face education have been reported to increase screening rates in general population [23]. However, little is known about whether these interventions of increasing appropriate knowledge could lead to increased SPC screening among cancer survivors. Furthermore, an interventional trial using educational materials to increase knowledge about SPC screening reported no increase in actual cancer screening for cancer survivors [24].

The present study showed that a lack of recommendation for SPC screening from physicians might have a negative impact on the colorectal and stomach cancer screening behaviors among lung cancer survivors. Similarly, a previous study reported that cervical cancer survivors who received, to whom their health care providers had recommended other cancer screening, were more likely to receive breast cancer screening [25]. After experiencing cancer, survivors usually have high levels of trust in their physicians, [26, 27] and physicians’ advice for screening might provide good opportunities to improve SPC screening behaviors.

Only 40.7% of disease-free lung cancer survivors, however, recalled being informed about the need for SPC screening or referred for such tests. Together with the results of previous studies, our results suggests that more information and training regarding appropriate cancer screening guidelines for cancer survivors will be needed for health care providers. Because there were few guidelines regarding such specific SPC screening, a feasible step should be started with increasing cancer survivors’ compliance to cancer screening guidelines for the general population. If physicians’ recommendation for SPC screening would be incorporated in the survivorship care plan, it might foster physician communication and shared care in monitoring SPC screening for cancer survivors.

We also found educational disparities in stomach and colorectal cancer screening among lung cancer survivors, and found income disparities in colorectal cancer screening among female subjects. Although several studies have reported educational and income disparities in cancer screening practices among the general population, [28, 29] little is known about these disparities among cancer survivors. In order to provide equal access to SPC screening services for cancer survivors, further collaborative efforts by policy makers, third party payers, and healthcare providers are needed. Several previous studied have suggested that educational disparity on receipt of cancer screening might be mediated by the role of health literacy [30, 31]. Further study for low-educated cancer patients will be needed to increase appropriate knowledge and attitude for SPC screening during or after the cancer treatment periods. Furthermore, because cancer survivors are more financially vulnerable, [32, 33] decreasing economic barrier for SPC screening should be considered.

Our study had several limitations. First, we used self-reported survey to assess the cancer screening compliance and physicians’ advice for SPC screening, which were not confirmed by medical record reviews or claims. Second, the response rate was only 44.2%. As participants could have been more likely to have preventive health behaviors than non-participants, our estimates of SPC screening practices among lung cancer survivors might therefore have been overestimated. Third, our study population consisted of Korean lung cancer survivors and stomach cancer screening is not recommended in western countries, which may limit the generalizability of our results. Although recent study has demonstrated that those who received an upper endoscopy were less likely to die from stomach cancer within the Korean national cancer screening program, [34] and cancer survivors were usually recommended to receive routine cancer screening guideline which is recommended in general population, [11–13] further evidences will be needed among other ethnicities.

## Conclusion

The present study showed that only a quarter of lung cancer survivors included were meeting existing guidelines for second primary cancer screening, particularly gastric and colorectal cancer. Physician must more proactive in communicating the need for screening and referring patients for such screening tests. In addition, further public policy will be needed to decrease educational disparities in SPC screening practices.

## Additional files

Additional file 1: Questionnaires about uptake of gastrointestinal cancer screening among lung cancer survivors. (DOCX 14 kb)

Additional file 2: Table S1. Characteristics of the participants and non-participants. (DOC 27 kb)

## Abbreviations

aOR: Adjusted odds ratios
CI: Confidence interval
SPC: Second primary cancer
