# Supplementary material for: Factors related with colorectal and stomach cancer screening practice among disease-free lung cancer survivors in Korea
Source: BMC Cancer. 2017 Aug 30;17:600. doi: 10.1186/s12885-017-3583-z (PMC5577681; doi:10.1186/s12885-017-3583-z)
Supplement: Supplementary file 1 — Questionnaires about uptake of gastrointestinal cancer screening among lung cancer survivors. (DOCX 14 kb) [file 12885_2017_3583_MOESM1_ESM.docx]

**Questionnaires about uptake of gastrointestinal cancer screening among lung cancer survivors**

**B1.** When did you receive a gastroscopy or double-contrast upper gastrointestinal series recently?

□ No □ ≤ 2 years ago □ 2–5 years ago □ >5 years ago

**B2.** What kind of colorectal cancer screening test did you receive?

□ no □ fecal occult blood test (FOBT)

□ double-contrast barium enema □ sigmoidoscopy □ colonoscopy

**B2-1.** If you receive a colorectal cancer screening, when did you receive the last colorectal cancer screening test?

□ No □ ≤ 1 years ago □ 1–5 years ago

□ 5–10 years ago □ >10 years ago

**B3.** Did you receive a physicians’ recommendation to screen for SPC after lung cancer treatment?

□ Yes □ No

**B4.** What do you approximate your risk of second primary cancer compared with cancer risk in general population?

□ Lower □ Similar □ Higher
