# Supplementary material for: Factors related with colorectal and stomach cancer screening practice among disease-free lung cancer survivors in Korea
Source: BMC Cancer. 2017 Aug 30;17:600. doi: 10.1186/s12885-017-3583-z (PMC5577681; doi:10.1186/s12885-017-3583-z)
Supplement: Supplementary file 2 — Characteristics of the participants and non-participants. (DOC 27 kb) [file 12885_2017_3583_MOESM2_ESM.doc]

**Table S1. Characteristics of the participants and non-participants**

| Characteristics | Participants | Non-participants | P |
| --- | --- | --- | --- |
| **Age, years** |  |  |  |
| <55 | 18.9% | 22.2% |  |
| ≥ 55 | 81.1% | 77.8% | 0.10 |
| **Gender** |  |  |  |
| Male | 76.8% | 71.9% |  |
| Female | 23.3% | 28.1% | 0.24 |
| **Residential area** |  |  |  |
| Metropolitan | 42.1% | 29.4% |  |
| City/Country | 57.9% | 70.6% | <0.01 |
| **Stage** |  |  |  |
| 0-I | 63.4% | 63.0% |  |
| II-III | 36.6% | 37.0% | 0.88 |
| **Hospital** |  |  |  |
| A hospital | 60.0% | 58.4% |  |
| B hospital | 40.0% | 41.6% | 0.51 |
